# Verticalization with Erigo® in early rehabilitation in patients with severe acquired brain injury: evidence from a pilot study

**DOI:** 10.3389/fresc.2026.1760006

**Published:** 2026-07-10

**Authors:** Valentina Colombo, Bledi Shehaj, Francesca Cesira Cava, Alice Rita Portillo, Ilaria Sermasi, Rossana Di Staso, Valerio Sveva, Francesca Serafino, Pamela Salucci

**Affiliations:** 1 Montecatone Rehabilitation Institute, Imola, Italy; 2 Department of Biomedical and Neuromotor Sciences, Alma Mater Studiorum University of Bologna, Bologna, Italy

**Keywords:** muscle hyperactivity, rehabilitation, robotics, severe acquired brain injury, verticalization

## Abstract

**Introduction:**

Following severe acquired brain injury (sABI), the inability to maintain orthostatism and prolonged bed rest triggers neurovegetative, haemodynamic, respiratory and osteo-myo-articular adaptations that may significantly affect rehabilitation. Restoring orthostatic tolerance is a key rehabilitation goal to prevent secondary complications. Although the safety of early verticalization with Erigo device has been established, evidence on its effects on muscle hyperactivity, trophism, and consciousness level in the subacute sABI remains limited. This pilot study aimed to monitor lower-limbs hyperactivity in patients with sABI of any aetiology, 1–4 months after the acute event, undergoing verticalization with the Erigo device in addition to conventional rehabilitation. Secondary aims were to evaluate muscle ultrasound characteristics, ankle mobility, disability, arousal level and device tolerability.

**Methods:**

All patients with early-stage sABI, admitted to the Critical Care Area of the Montecatone Rehabilitation Institute from March 2022 to April 2024, were included. Each patient underwent 10 sessions of approximately 30 min, once a day, 3–5 times per week. Assessments before and after treatment included electroneurography of the lower limbs, muscle ultrasound measurement of the tibialis anterior and medial twin muscles, bilaterally, and rating scales evaluating disability and arousal (Disability Rating Scale - DRS, Levels of Cognitive Functioning - LCF, Coma Recovery Scale Revised - CRS-R). Adverse events related to device were monitored daily.

**Results:**

The study includes 22 patients with sABI. Neurophysiologically, there was a reduction or invariance in the H max/M max ratio compared to the initial assessments; the Heckmatt scale scores remained unchanged in all patients. All the mobility measures, the H-reflex amplitude, the Hmax/Mmax ratio and the MAS scores remained stable. The functional measures improved significantly: CRS-R (*p* = 0.005), LCF (*p* = 0.002) and DRS (*p* < 0.001).

**Discussion:**

This pilot study suggests that early verticalization with Erigo is a feasible and well-tolerated intervention in patients with sABI in the subacute phase of rehabilitation. No worsening of muscle hyperactivity or muscle ultrasound characteristics was observed during the intervention period. A significant improvement in cognitive function was observed. Controlled studies are required to determine whether these findings are attributable to the Erigo intervention.

## 1 Introduction

Severe Acquired Brain Injury (sABI) is defined as a damage to the brain, occurring from traumatic brain origin or non-traumatic causes (such as ischemic or haemorrhagic stroke, anoxia following cardiac arrest), with a coma state lasting at least 24 h and leading to disability (1). Approximately 214,000–230,000 individuals are hospitalized annually for traumatic brain injuries (TBIs). Because exact clinical severity is a spectrum, global figures include both moderate and severe cases; in the European Union alone, over 1 million people are admitted to hospitals for moderate or severe brain injuries every year (2). Patients with more severe symptoms frequently do not regain complete consciousness. However, these patients often enter a phase of prolonged or persistently altered state of consciousness (Disorder of Consciousness, DoC) such as Vegetative State (VS) or Minimally Conscious State (MCS) (3). In particular, the Level of Cognitive Functioning (LCF) scale can be used to distinguish between patients with a protracted low level of responsiveness and slow recovery (LCF ≤ 3), and patients who have recovered an almost constant level of interaction with the environment but manifest various degrees of cognitive and behavioural impairment (LCF > 4) (4). Patients with sABI are generally complex cases, presenting sensory-motor impairments in addition to cognitive-behavioural disorders that impact all phases of rehabilitation treatment involving an interdisciplinary team. The rehabilitation objectives in the earliest stages of treating these patients include improving respiratory function, restoring vasomotor control, preventing pressure injuries, and promoting postural changes to prevent complications from prolonged postures. These may include muscle-tendon shortening, reduced joint mobility, and altered muscle tone. In patients with sABI, the inability to maintain upright posture and the prolonged bed rest induce a cascade of autonomic, haemodynamic, and respiratory adaptations, together with significant musculoskeletal alterations. These secondary changes exert a significant impact on long-term prognosis (5, 6). Verticalization contributes to the re-establishment of vasomotor regulation, optimisation of respiratory mechanics, prevention of osteoporosis and pressure injuries, attenuation of disuse atrophy and spasticity, and enhancement of trunk postural control. Passive verticalization using a static tilt table is a well-established intervention in intensive neurorehabilitation settings. However, the use of a conventional static tilt table may be limited by orthostatic hypotension resulting from reduced cardiac output due to lower-limb venous pooling and/or concomitant impairment of the central sympathetic system. Among the technological devices to facilitate the rehabilitation process, there is the Erigo robotic tilt table (Hocoma, Volketswill, Switzerland). Erigo assists verticalization up to 90° in a gradual and controlled manner, concomitantly enabling rhythmic movement of the lower limbs that simulates walking. This treatment also facilitates contact and interaction with the surrounding environment for the patient (7, 8). Emerging rehabilitation technologies enable high-intensity, task-specific training while also allowing a more standardized and reproducible rehabilitation approach. In patients with sABI, robotic devices have been employed to facilitate early verticalization, improve trunk control, support gait rehabilitation, and enhance upper-limb recovery (9–12). Among these technologies, the Erigo robotic tilt table combines gradual verticalization with passive stepping movements, improving venous return and cardiovascular adaptation during mobilization. Unlike conventional static tilt tables, which provide passive standing without lower-limb activation, Erigo integrates gradual verticalization with cyclic robotic stepping and repetitive limb loading, thereby promoting venous return and reducing orthostatic intolerance during mobilization (7, 13). This combined approach allows early mobilization while minimizing the cardiovascular instability frequently observed during conventional passive verticalization in critically ill neurological patients. More broadly, robot-assisted rehabilitation systems in patients with severe acquired brain injury (sABI) have been increasingly developed to deliver intensive, repetitive, and task-specific training while ensuring high reproducibility and standardized treatment parameters. Depending on the patient's level of residual motor and cognitive function, different robotic technologies may target trunk control, gait recovery, upper-limb rehabilitation, or early mobilization. However, many robotic gait trainers and wearable exoskeletons generally require active participation, residual trunk control, or a certain degree of voluntary motor function, thus limiting their applicability during the earliest stages of recovery or in patients with disorders of consciousness.In contrast, Erigo can also be employed in severely impaired or minimally conscious patients during the subacute and early rehabilitation phases, when active participation is still limited (7, 14). The integration of robotic stepping with gradual orthostatic training may facilitate multisensory stimulation, improve afferent proprioceptive input, and promote interaction with the environment while maintaining clinical safety. Furthermore, the integrated monitoring systems allow continuous supervision of cardiovascular tolerance and patient stability throughout treatment sessions, making the device particularly suitable for critically ill patients with severe neurological impairment. These characteristics position Erigo as a bridge between passive mobilization strategies and more advanced active robot-assisted gait rehabilitation technologies. Recent systematic reviews and meta-analyses demonstrated that Erigo represents a safe and feasible intervention for early mobilization in neurological patients and may reduce the incidence of orthostatic hypotension (9). It also appears to reduce spasticity as measured by the Modified Ashworth Scale (MAS) (15), whereas its effects on muscle strength (9, 15), functional recovery (8), and level of consciousness in patients with sABI remain uncertain (7, 14, 16). Although clinical scales remain the standard assessment tools in patients with sABI, recent studies have increasingly explored the use of quantitative neurophysiological and sensor-based biomarkers to obtain more objective measures of neurological recovery and patient performance. Quantitative EEG-derived biomarkers may provide objective information regarding cortical connectivity, residual cognitive processing, neural network integrity, and level of consciousness, potentially allowing the detection of subtle neurophysiological changes not always identifiable through behavioural observation alone. Similarly, sensor-based rehabilitation systems may continuously monitor motor performance, symmetry, loading patterns, and physiological adaptation during treatment sessions (17–20). Nevertheless, despite their inherent categorical nature and limited sensitivity to subtle inter-subject variability, validated behavioural scales such as DRS, LCF, and CRS-R (21, 22) remain the current clinical reference standard in patients with sABI because they are reproducible, widely adopted in neurorehabilitation practice, directly associated with functional prognosis, and highly relevant for rehabilitation planning and longitudinal clinical monitoring. The integration of these advanced assessment approaches with robotic rehabilitation technologies may represent an important future direction in this field. Considering the safety of the device when used early in patients with sABI and the uncertainty surrounding the efficacy of Erigo in certain outcomes, this pilot study was conducted with the objective to obtain a preliminary estimate of the effect of 10 Erigo sessions on functional and mobility outcomes. The specific outcome measures encompassed spasticity, muscle ultrasound characteristics, ankle joint mobility, muscle hyperactivity, degree of disability, cognitive level, and level of consciousness.

## 2 Related works

Several studies have investigated the use of robotic verticalization devices in patients with severe neurological conditions, with particular attention to safety, orthostatic tolerance, spasticity, and functional recovery (Table 1). Frazzitta et al. (7) conducted a randomized pilot study in ICU patients with sABI, demonstrating that early stepping verticalization with Erigo was feasible and associated with improvement in LCF scores. Ancona et al. (8), reported significant improvements in Coma Recovery Scale- Revised (CRS-R) scores following Erigo treatment in patients with acute sABI, confirming the safety of the device in this population. Krewer et al. (16), investigated tilt table therapies in patients with severe disorders of consciousness, finding no significant changes in CRS-R, and highlighting the need for more sensitive outcome measures. Kumar et al. (14), compared Erigo with conventional physiotherapy in acute stroke patients, reporting superior spasticity reduction in the robotic group as measured by MAS. A recent systematic review and meta-analysis by Garlet et al. (9), confirmed that Erigo significantly reduces the incidence of orthostatic hypotension and spasticity across neurological populations, whereas the effects on muscle strength and functional recovery remain uncertain. Compared with these works, the present study adopts a multimodal assessment approach that combines neurophysiological measures (Hoffmann reflex, Hmax/Mmax ratio), evaluation of muscle ultrasound characteristics (Heckmatt scale), ankle joint mobility, and clinical scales, providing a more comprehensive characterization of the effects of early verticalization in the subacute sABI population.

**Table 1:** Overview of related works on robotic verticalization in neurological rehabilitation.

Study	Device	Population	N	Outcome measures	Main findings
Frazzitta et al. (7)	Erigo	sABI (ICU)	20	CRS-R, GCS, LCF, DRS	Feasible; CRS-R improvement
Ancona et al. (8)	Erigo	sABI (acute)	30	NIHSS, Tinetti, FIM	Safety; CRS-R improvement
Krewer et al. (16)	Tilt table	DoC	34	CRS-R	No significant CRS-R change
Kumar et al. (14)	Erigo	Stroke (acute)	40	SF-36, MRC, NIHSS, MMSE, MAS	QoL, strenght and spasticity improvement
Garlet et al. (9)	Erigo	Mixed neurological	Meta-analysis	MAS, MRC, FIM, CRS-R, LCF	Safety, Reduced spasticity
Present study	Erigo	sABI (subacute)	22	Hmax/Mmax, Heckmatt scale, CRS-R, LCF, DRS, MAS	Stable hyperactivity; functional improvement

sABI, severe acquired brain injury; GCS, glasgow coma scale; LCF, levels of cgnitive functioning; MAS, modified ashworth scale; CRS-R, coma recovery scale-revised; DRS, disability rating scale; NIHSS, national institutes of health stroke scale; FIM, functional independence measure; DoC, disorder of consciousness; SF-36, short form-36 health survey; QoL, quality of life.

## 3 Materials and methods

### 3.1 Study design and setting

This is an observational, prospective, single-centre pilot study carried out in the Intensive and Sub-Intensive Care Unit and the ABI Rehabilitation Unit at the Montecatone Rehabilitation Institute Hospital (Imola, Bologna, Italy) by a multidisciplinary team composed of physiatrists, physiotherapists and neurophysiopathology technicians.

### 3.2 Subjects

Between March 2022 and April 2024, 28 consecutive patients were enrolled in this study within 4 months from a severe ABI. 22 patients actually completed the Erigo treatment. Patients were identified by the Unit physiatrist after evaluation of enrolment criteria.

Inclusion criteria were:

- age 18–74;
- time since acute neurological event between 1 and 4 months;
- diagnosis of sABI of any aetiology (vascular, traumatic, anoxic, infective) admitted in ordinary care at Montecatone Rehabilitation Institute, Imola (Bologna, Italy);
- cardio-pulmonary and circulatory stability.

Exclusion criteria were:

- previous other sABI;
- recovery of autonomous or with movable support orthostatism and/or of walking at the admission to the Institute;
- delayed unions/ non-unions limbs fractures or vertebral instability, severe osteoporosis;
- severe heart failure and cardiovascular diseases;
- non-adaptable constitutions (leg length < 75 cm or >100 cm; weight > 135 kg)
- irreducible leg contractures or grade > 3 hypertonia according to the Modified Ashworth Scale (MSA);
- opposition or aggressiveness;
- current pregnancy.

All patients signed an informed consent to participation in the study. If they were incapable, their representative had to sign it on their behalf.

### 3.3 Equipment

Erigo is a rehabilitation robotic device designed for the early mobilization and verticalization of severely impaired patients. The Erigo combines gradual verticalization with cyclic leg movements and loading for the stabilization of the patient in the upright position. The key features are: progressive verticalization up to 90°, cyclic leg movements 8–80 steps/min, adjustable guidance force for leg drives 0%–100% (symmetric/asymmetric), adjustable range of motion 0–45° (symmetric/asymmetric), adjustable leg loading with integrated real-time feedback of the loading force, different movement patterns (sine wave, gait, alternating leg) and FES stimulation synchronized with the leg movement of the device, if used.

### 3.4 Treatment protocol

The treatment protocol consisted of single daily sessions of verticalization using a tilt table with an integrated robotic stepping device Erigo (Hocoma AG, Volketswil, Switzerland) located in the Gym of the Intensive and Sub-Intensive Care Unit. Physiotherapists specializing in neurologic rehabilitation prepared the patients and supervised treatment with Erigo, while an intensive care doctor was available in case of emergency. They had been specially trained in the correct use of the device at the time of purchase. During treatment, the patient's upper body was secured to the table using a harness that fastened around the chest and shoulders. Their feet were strapped to two footplates, and their distal thighs were secured to a stepping device. The patient's legs performed passive stepping movements, which were achieved through the rhythmic, alternating pushing up of the feet, controlled by a computer. Although the Erigo device includes an optional functional electrical stimulation (FES) module for the lower limbs, this feature was intentionally not activated in the present protocol. The rationale was to isolate the effects of passive verticalization and robotic stepping without the confounding influence of electrically induced muscle contractions, thereby allowing a clearer interpretation of outcomes related to muscle hyperactivity and trophism. The session lasted 30 min in total, not including the time required for transferring the patient and setting up the machine. After patient positioning, the slope of the tilt table was gradually increased from 0° to 90°. The majority of subjects demonstrated an average tolerance for verticalization ranging from 60° to 80°. The stepping frequency was not set at a constant level during all sessions, but varied from a minimum of 20 steps/min to a maximum of 40 steps/min. Monitoring of cardiovascular and respiratory parameters was conducted at the beginning and end of treatment. Sessions were performed 3–5 times per week until a total of 10 sessions per patient. In addition to Erigo treatment, patients received daily conventional physiotherapy (exercises to maintain and restore muscle length, exercises to improve upper limb or trunk function depending on the clinical situation) and other speech therapy, neuropsychology and occupational therapy treatments 2–3 times a week.

### 3.5 Outcome measures

The following assessments were conducted at the enrolment in the study, and after the conclusion of the rehabilitation treatment period with Erigo:

- measurement of ankle joint mobility;
- measurement of spasticity of the lower limb muscles with Modified Ashworth Scale (MAS) (15);
- measurement of the tibial nerve H reflex amplitude with stimulation at the level of the soleus muscle;
- measurement of the H max/M max ratio;
- measurement of the stimulus intensity threshold;
- muscle ultrasound measurement with a linear probe of the tibialis anterior and medial twin muscles, bilaterally (Heckmatt scale) (23);
- Disability Rating Scale – (DRS) (24);
- Levels of Cognitive Functioning – (LCF);
- Coma Recovery Scale Revised – (CRS-R) (21, 22).

The Heckmatt ultrasonographic scale (23) is a semi-quantitative method that assesses muscle structural quality by grading echogenicity and the visibility of the bone echo beneath the muscle. In particular:

- Grade 1 represents normal muscle architecture with clear visibility of the bone echo.
- Grade 2 indicates a slight increase in echogenicity, but the bone echo remains well defined.
- Grade 3 shows marked echogenicity with partial loss of the bone echo, suggestive of intramuscular fibrosis or fatty infiltration.
- Grade 4 is characterized by very high echogenicity and complete loss of the bone echo, consistent with severe fibrotic replacement of contractile tissue.

Higher grades therefore reflect a greater degree of structural alteration, often due to denervation, chronic spasticity, or immobilization.

From a neurophysiological perspective, the H-reflex (Hoffmann reflex) provides an objective measure of spinal excitability. It reflects the monosynaptic activation of *α*-motoneurons through Ia afferent fibers from muscle spindles. The Hmax/Mmax ratio represents the proportion of the motoneuron pool that can be activated reflexively compared with direct activation by electrical stimulation, serving as an indicator of motoneuronal excitability (25–27). Neurophysiological assessments were performed by the same trained neurophysiopathology technician at both the pre- and post-treatment time points, in order to minimize inter-rater variability. All recordings were obtained using a standard electromyography (EMG) system, with the patient in a standardized prone position, lower limb slightly flexed at the popliteal fossa (approximately 150°) and at the ankle (approximately 100°), with the foot projecting beyond the edge of the examination table. Electrode placement. Surface recording electrodes were positioned over the soleus muscle (active electrode placed just medial to the midline, halfway between the popliteal fossa and the Achilles tendon) and over the Achilles tendon (reference electrode). A ground electrode was placed between the stimulation and recording sites. The tibial nerve was stimulated at the popliteal fossa using a bipolar surface electrode (cathode proximal, anode distal), which represents the standard configuration for tibial H-reflex recording (25). Stimulation parameters. Rectangular pulses of 1 ms duration were delivered at gradually increasing intensities (0.1 mA increments), starting below motor threshold, in accordance with standard H-reflex methodology (25). Band-pass filter settings were: low-frequency filter (LFF) 2–20 Hz, high-frequency filter (HFF) 5–10 kHz; recommended sensitivity 0.5–1 mV. Determination of Hmax and Mmax. Stimulation intensity was increased stepwise to construct the full H-reflex/M-wave recruitment curve. The Hmax/Mmax ratio was calculated as the ratio between the peak-to-peak amplitude of the maximum H-reflex and the peak-to-peak amplitude of the maximum M-wave. This ratio provides an index of the proportion of the motoneuron pool that can be reflexively activated and is widely used as a neurophysiological marker of spinal motoneuron pool excitability and spasticity in patients with upper motor neuron lesions (25–27).

### 3.6 Statistical analysis

An *a priori* power analysis was performed using G*Power version 3.1.9.4 for the Wilcoxon signed-rank test. The calculation was based on the improvement observed in Level of Cognitive Functioning (LCF) scores reported by Frazzitta et al. (7) in patients with severe acquired brain injury treated with Erigo. Assuming a medium effect size (Cohen's d = 0.5), an alpha level of 0.05, and a statistical power of 80%, a minimum sample size of 28 participants was required.

Categorical variables were described as absolute and percentages frequencies, whereas continuous variables were described as mean and standard deviation, or median and interquartile range. Shapiro–Wilk's test was carried out to determine the normality of the frequency distribution.

The non-parametric Wilcoxon test was used to compare scale scores recorded before treatment and after 10 sessions. For variables that showed a statistically significant change in scores compared to baseline, regression models were estimated to assess the effect of age, gender, BMI, CIRS and hemorrhagic aetiology on outcomes after 10 sessions, adjusted for the same scores recorded before the start of treatment.

Linear regression models were constructed for the CRS, LCF and DRS variables, whereas a Poisson model was constructed for the right hip MAS ADD variable, as the assumptions of normality did not hold. Each regression model included one predictor, and was adjusted for the baseline score of the outcome measure.

For all analyses, the significance level was set at *p* < 0.05.

## 4 Results

**Figure 1:**
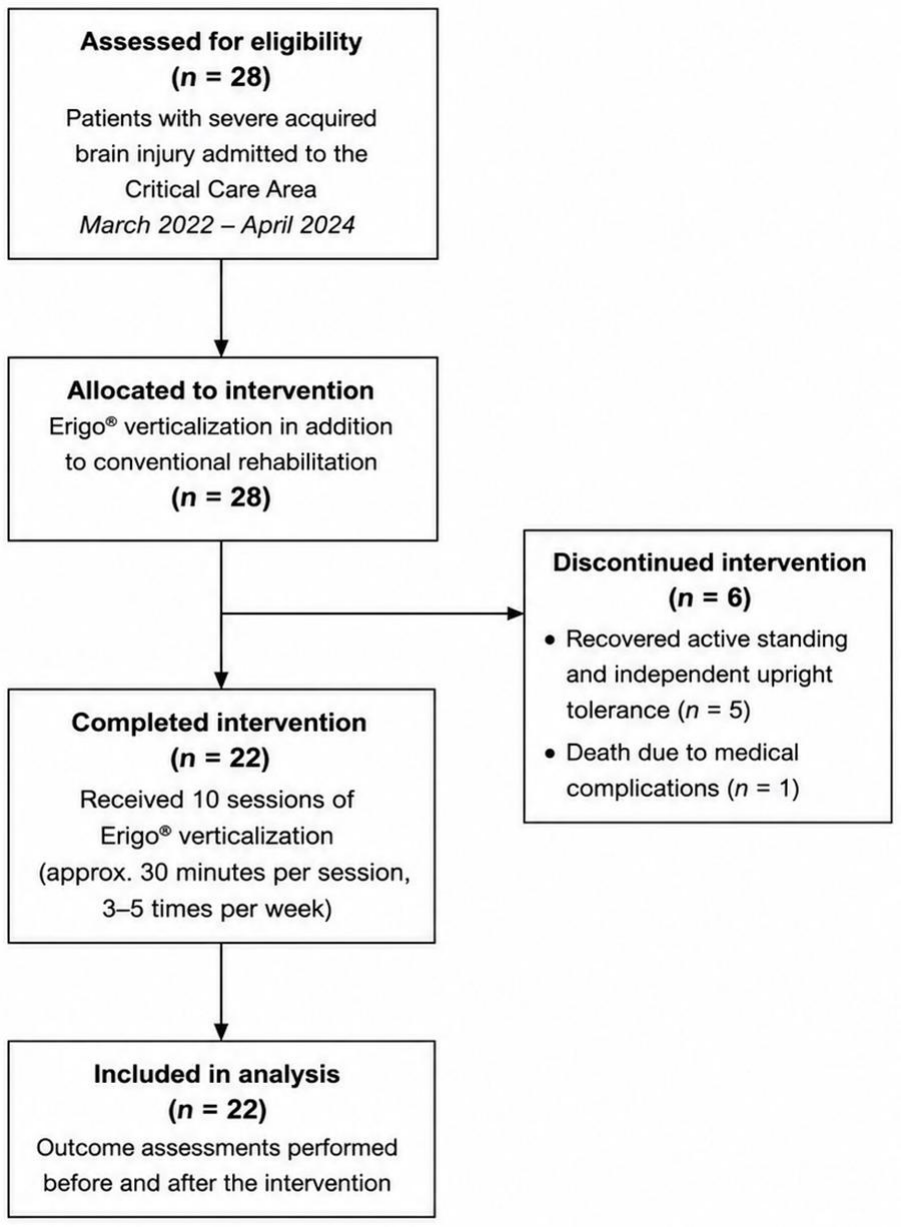
Flow diagram of patient enrollment and study completion.

Of the 28 patients enrolled, 22 completed the treatment protocol (see Figure 1). Five patients were withdrawn from the protocol because they developed active orthostatism during treatment and therefore no longer met the device criteria. One patient died from causes unrelated to the intervention. A comparison of baseline demographic and clinical characteristics between completers and non-completers is provided in Table 2. Moreover, there were no significant differences in age, sex, BMI, CIRS score or etiology between groups, suggesting that attrition is unlikely to have introduced a selection bias.

**Table 2:** Baseline demographic and clinical characteristics of patients who completed the Erigo® treatment protocol (completers, *N* = 22) versus patients who did not complete the protocol (non-completers, *N* = 6).

Variable	Completers (*N* = 22)	Non-completers (*N* = 6)
Sex, male, N (%)	16 (73%)	3 (50%)
Age, years, mean (SD)	55.0 (11.6)	56.8 (18.4)
BMI, mean (SD)	23.7 (1.4)	21.9 (3.3)
CIRS score, mean (SD)	1.04 (1.43)	2.0 (1.2)
Time since injury, months, median (IQR)	1–4	1–4
Aetiology, N (%)		
Haemorrhagic	11 (50%)	2 (33%)
Traumatic	5 (23%)	3 (50%)
Anoxic	5 (23%)	1 (17%)
Neoplastic	1 (4%)	0 (0%)
Reason for non-completion, N (%)		
Recovered active orthostatism	—	5 (83%)
Death (unrelated to intervention)	—	1 (17%)

BMI, body mass index; CIRS, cumulative illness rating scale; IQR, interquartile range; N, number; n.a, not available; SD, standard deviation.

Of the 22 completers, 16 were men (73%). Mean age was 55 years (SD = 11.6), mean BMI was 23.7 (SD = 1.4) and the mean CIRS score was 1.04 (SD = 1.43). Etiology was hemorrhagic in 11 (50%) patients, traumatic in 5 patients, anoxic in 5 patients and neoplastic in 1 patient. Table 3 shows the descriptive statistics and the results of Wilcoxon tests comparing the pre-post assessments of Erigo tests and of functional outcomes.

**Table 3:** Scores of ERIGO tests and functional outcomes before and after 10 treatment sessions.

		Before erigo (t0)			After erigo (t1)			comparison t0-t1	
Variable		mean	SD	N (%)	mean	SD	N (%)	V^a^	p
MAS F hip	right	0.45	0.86		0.45	0.8		1.5	1
	left	0.36	0.73		0.41	0.67		2	0.77
MAS E hip	right	0.23	0.61		0.41	0.8		0	0.34
	left	0.14	0.47		0.27	0.63		0	0.37
MAS ADD hip	right	0.18	0.59		0.5	0.86		0	**0**.**04**
	left	0.36	0.73		0.64	0.85		6	0.19
MAS F knee	right	0.41	0.80		0.36	0.79		3.5	1
	left	0.27	0.63		0.36	0.73		1.5	0.59
MAS E knee	right	0.36	0.80		0.32	0.72		2	1
	left	0.31	0.65		0.23	0.53		4.5	0.59
MAS F footbed	right	0.09	0.43		0.14	0.47		0	1
	left	0.09	0.42		0.09	0.43		0	NA
MAS INV ankle	right	0.36	0.85		0.32	0.78		9	0.78
	left	0.41	0.85		0.36	0.79		9	0.78
ROM F footbed	right	41.36	3.51		39.77	9.57		2	1
	left	41.36	3.51		38.41	11.48		5	0.42
ROM F backbone	right	10.45	4.61		10.91	5.48		5	0.57
	left	9.54	5.32		10.68	5.62		5	0.28
									
CRS-R		7.61	5.57		9.54	5.63		0	**0**.**005**
	not applicable			7 (32)			13 (50)		
LCF		3.45	1.41		4.18	1.53		0	**0**.**002**
DRS		20.5	5.32		17.59	6.29		136	**0**.**0004**
HECK TA		1.54	0.67		1.61	0.66		0	1
HECK GM		1.59	0.67		1.68	0.65		0	0.35
pain	yes			5 (22)			1 (5)		
	no			17 (78)			21 (95)		
H reflex	right	3.18	2.85		3.97	3.7		50	0.13
	not applicable			3 (11)			4 (18)		
	left	3.10	2.80		3.39	3.04		76	0.3
	not applicable			1 (4)			1 (4)		
Hmax/Mmax	right	0.35	0.20		1.82	6.1		62	0.32
	not applicable			3 (11)			4 (18)		
	left	0.33	0.18		0.42	0.42		77	0.31
	not applicable			1 (4)			1 (4)		
threshold stimulus	right	9.64	4.93		10.94	5.13		21	0.17
	not applicable			3 (11)			4 (18)		
	left	11.44	5.09		10.95	5.67		67	0.36
	not applicable			1 (4)			1 (4)		

CIRS, cumulative illness rating scale; BMI, body mass index; MAS Modified Ashworth Scale; F, flexor; E, extensor; ADD, adductors; INV, inversor; ROM, range of motion; CRS-R, coma recovery scale-revised; LCF, levels of cognitive functioning; DRS, disability rating scale; HECK, Heckmatt Scale; TA, tibialis anterior muscle; GM, medial twin muscle; H reflex, Hoffmann reflex; SD, standard deviation.

a Wilcoxon signed rank test for paired data.

Bold values indicate statistically significant differences between pre- and post-treatment assessments (*p* < 0.05).

All the mobility measures, the H-reflex amplitude, the Hmax/Mmax ratio and the MAS scores remained stable except for MAS hip adductor (ADD) right that showed a mild but significant worsening after 10 Erigo sessions (*p* = 0.04).

**Figure 2:**
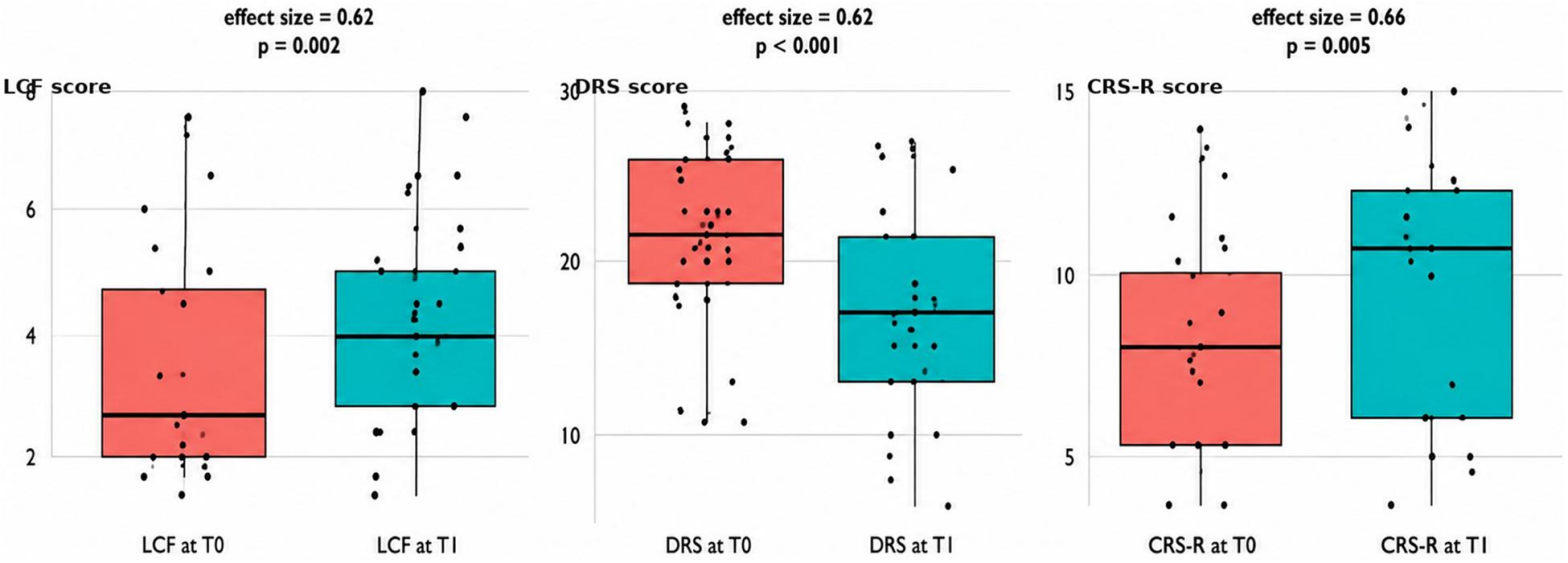
Baxplots showing the improvement of functional outcomes from baseline to the end the 10 Erigo treatment sessions. Significant increases are found in the level of agnitive function (LCF), *p* = 0.002, and in the total score of CRS-R (*p* = 0.005) Significant decreases are observed in the disability level (DRS). *p* < 0.001.

The functional measures improved significantly: CRS-R (effect size=0.96, *p* = 0.005), LCF (effect size=0.82, *p* = 0.002) and DRS (effect size=0.83, *p* = 0.0004) (Table 4 and Figure 2). No patient had adverse events and 14% had complications, including orthostatic hypotension (*N* = 1) or pain (*N* = 2).

**Table 4:** Results of regression models, showing the effect of demographic and clinical variables on outcomes results are expressed as regression coefficients (b). the standard deviation of the coefficients [sd(b)] and their significance (p). The effects of age, sex, BMI, CIRS and etiology are investigated in models 1–5, adjusted for the baseline score of the outcome.

	DEPENDENT VARIABLE											
	MAS ADD hip^a^			CRS-R^b^			LCF^b^			DRS^b^		
	b	sd(b)	p	b	sd(b)	p	b	sd(b)	p	b	sd(b)	p
MODEL 1												
baseline value	1.27	0.43	**0** **.** **003**	1.19	0.17	**<0**.**0001**	0.90	0.14	**<0**.**0001**	0.98	0.15	**<0**.**0001**
age	0.10	0.05	**0**.**04**	−0.02	0.04	0.59	−0.003	0.02	0.82	−0.01	0.07	0.90
MODEL 2												
baseline value	0.91	0.32	**0**.**005**	1.19	0.17	**<0**.**0001**	0.88	0.15	**<0**.**0001**	0.93	0.15	**<0**.**0001**
sex (M)	0.34	0.70	0.62	−0.18	1.23	0.88	0.19	0.45	0.69	−2.14	1.76	0.24
MODEL 3												
baseline value	1.06	0.38	**0**.**006**	1.20	0.18	**<0**.**0001**	0.94	0.14	**<0**.**0001**	1.01	0.16	**<0**.**0001**
BMI	0.08	0.09	0.32	0.034	0.21	0.87	−0.05	0.05	0.29	0.11	0.19	0.59
MODEL 4												
baseline value	0.87	0.32	**0**.**006**	1.19	0.16	**<0**.**0001**	0.91	0.14	**<0**.**0001**	1.00	0.15	**<0**.**0001**
CIRS	0	0.21	1	−0.42	0.40	0.32	0.09	0.14	0.53	−0.56	0.55	0.32
MODEL 5												
baseline value	0.87	0.31	**0**.**005**	1.18	0.17	**<0**.**0001**	0.93	0.15	**<0**.**0001**	1.02	0.16	**<0**.**0001**
haemorrhagic etiology	0.18	0.60	0.76	−0.44	1.21	0.72	−0.30	0.41	0.47	1.18	1.68	0.49

CIRS, cumulative illness rating scale; BMI, body mass index; MAS, Modified Ashworth Scale; ADD, adductors; CRS-R, coma recovery scale-revised; LCF, levels of cognitive functioning; DRS, disability rating scale.

a Poisson regression.

b Linear regression.

Bold values indicate statistically significant differences between pre- and post-treatment assessments (*p* < 0.05).

None of the demographic and clinical variables included in the regression models were associated with the functional outcomes. However, increasing age was associated with higher levels of spasticity in the right hip adductor muscles (MAS ADD hip right) at the end of treatment after adjusting for the baseline score of the scale (Table 3).

## 5 Discussion

The stability of muscle hyperactivity in patients with sABI suggests that early rehabilitation can help prevent deterioration and complications. In this population, prolonged immobility often leads to a rapid worsening of the neuromuscular condition, characterized by increased spasticity, contractures, and muscle stiffness. Early verticalization using the Erigo may have acted as a preventive measure by maintaining muscle tone within a stable range and preserving joint mobility. From this perspective, the absence of deterioration represents a clinically favorable outcome, even in the absence of direct improvement.

Although no significant changes were detected in the neurophysiological (H-reflex and Hmax/Mmax ratio) and ultrasonographic (Heckmatt scale) parameters, their stability can nevertheless be interpreted as a positive result. Increased values are typically associated with heightened reflex activity and spasticity, as seen in upper motor neuron lesions (26, 27). Therefore, the stability of the H-reflex and Hmax/Mmax ratio in this study suggests that spinal excitability did not increase during the treatment period — a desirable outcome in early rehabilitation, when an upward trend would otherwise be expected.

A statistically significant worsening of spasticity in the right hip adductor muscles (MAS ADD right, *p* = 0.04) was observed, while all other MAS measures remained stable. Several explanations may account for this finding. First, measurement variability inherent to the MAS - a semi-quantitative ordinal scale with known inter-rater variability - may have contributed to this isolated significant result. Second, asymmetric neurological involvement, which is common in sABI patients regardless of aetiology, may have resulted in differential progression of spasticity in specific muscle groups. Third, the small sample size increases type I error rather and may not reflect a genuine clinical trend. Finally, clinical progression of spasticity in hip adductors unrelated to the Erigo intervention cannot be excluded, as this muscle group is particularly susceptible to spastic hypertonia in patients with prolonged neurological lesions. This finding should be confirmed in future studies with larger samples and a control group.

From a biological standpoint, it has been established that immobility rapidly induces maladaptive changes in muscle connective tissue, including activation of profibrotic pathways, intramuscular fibrosis, and increased passive stiffness, all of which may contribute to spasticity and contracture development (28–31). The cyclic limb loading and passive stretching provided by Erigo may have counteracted some of these processes through mechanotransduction; however, this remains a hypothesis, as no direct biological or structural measures were obtained in the present study. Future studies incorporating muscle biopsy, shear-wave elastography, or quantitative ultrasound techniques could test these mechanistic hypotheses more rigorously.

The significant improvements observed in CRS-R, LCF, and DRS scores should be interpreted with caution. Patients were assessed between 1 and 4 months after the acute neurological event, a period that corresponds to the phase of most intense spontaneous neurological recovery following sABI, during which meaningful functional gains are expected regardless of any specific intervention. Furthermore, all patients simultaneously received conventional multidisciplinary rehabilitation, including physiotherapy, speech therapy, and occupational therapy, throughout the study period. Therefore, the observed improvements in functional and cognitive scales are consistent with the natural history of the condition and with the effects of the overall rehabilitation program, and cannot be attributed specifically to the Erigo intervention. The absence of a control group receiving conventional rehabilitation alone makes it impossible to disentangle the contribution of robotic verticalization from that of spontaneous recovery and concurrent therapies. These findings should accordingly be interpreted as preliminary and compatible with a potential additive role of early verticalization in a comprehensive rehabilitation program, rather than as evidence of a specific treatment effect. Controlled studies with an appropriate comparator arm are required to determine whether Erigo provides benefits beyond those attributable to spontaneous recovery and conventional multidisciplinary rehabilitation.

This pilot study suggests that early verticalization with Erigo is a feasible and well-tolerated intervention in patients with sABI in the subacute phase. The absence of serious adverse events and the stability of the monitored clinical parameters during the treatment period support short-term tolerability of the procedure. The gradual tilt, integrated stepping movement, and cardiovascular monitoring make the procedure well tolerated even in patients with severe neurological and systemic impairment.

This pilot study has several limitations, including the limited number of treatment sessions, which may have reduced the ability to detect changes in muscle or neurophysiological parameters. A further consideration concerns the six patients who did not complete the protocol. Although the reasons for non-completion were unrelated to the Erigo intervention (five patients recovered active orthostatism; one died), the relatively small final sample (*N* = 22) limits the generalizability of the findings. The regression analyses investigating the demographic and clinical factors associated with functional improvement should be considered exploratory and hypothesis-generating. Another important limitation is the absence of a control group undergoing conventional rehabilitation alone, which does not allow to disentangle the effect of Erigo intervention from that of conventional rehabilitation. Future controlled studies with age-matched comparison groups are needed to better define the specific contribution of robotic verticalization to functional recovery in patients with sABI. In addition, although cardiovascular and respiratory parameters were monitored during treatment sessions to ensure clinical stability, respiratory variables were not systematically analysed as outcome measures. Future studies should integrate quantitative respiratory assessments in order to better investigate the effects of early robotic verticalization on pulmonary adaptation and respiratory recovery. In addition, although standardized clinical scales remain the most widely adopted tools for assessing disorders of consciousness and cognitive recovery in patients with sABI, they are inherently observer-dependent and may not fully capture subtle neurophysiological changes (30, 32, 33). The present study did not include objective neurophysiological biomarkers such as electroencephalography (EEG)-based indices or sensor-based monitoring systems, which may provide more sensitive and continuous measures of recovery and treatment response. Moreover, the number of patients was lower than that required by the sample size calculation. However, the reason why six patients were not included in the final sample was unrelated to the Erigo intervention and does not constitute a source of bias. In addition, the effect size for the change in functional measures was consistently greater than 0.80.

Future studies should address the limitations of the present work through a multicenter randomized controlled trial design, incorporating: a control arm receiving conventional rehabilitation without Erigo; stratification by aetiology (traumatic, vascular, anoxic) to identify subgroups that may benefit most; longer follow-up periods (3–6 months post-treatment) to assess the durability of any effects; quantitative EEG-based biomarkers to provide objective, continuous measures of consciousness and cortical recovery alongside clinical scales; instrumented gait and mobility measurements derived from sensors embedded in the robotic device; a larger sample size, with a formal *a priori* power calculation based on the effect sizes reported in the present study.

### 5.1 Clinical implications

The findings of this pilot study have several practical implications for neurorehabilitation units managing patients with sABI. First, regarding patient selection, Erigo appears to be particularly suitable for patients in the subacute phase (1–4 months post-injury) who have not yet recovered active orthostatism and present with disorders of consciousness or minimal responsiveness, given the device's ability to provide graduated verticalization without requiring active patient cooperation. Second, regarding timing of initiation, the results support early initiation of robotic verticalization - within the first four months post-injury - as part of a comprehensive rehabilitation program aimed at preventing secondary complications of immobility. Third, from an organizational standpoint, the implementation of Erigo in a rehabilitation unit requires dedicated physiotherapist training, availability of emergency medical supervision during sessions, and appropriate patient selection to minimize the risk of orthostatic hypotension. The low rate of complications observed in this study (14%, all minor and self-resolving) suggests that the procedure is manageable within a standard intensive neurorehabilitation setting.

## Data Availability

The original contributions presented in the study are included in the article/Supplementary Material, further inquiries can be directed to the corresponding author.
